# Successful Treatment of a Refractory Lymphatic Fistula after Laparoscopic Para-Aortic Lymphadenectomy Using Dual Real-Time Lymphangiography with Indocyanine Green and a High-Fat Diet

**DOI:** 10.70352/scrj.cr.24-0183

**Published:** 2025-05-01

**Authors:** Tomoaki Okada, Akinari Nomura, Shinya Yamashita, Ryuichi Kita, Shun Akiyama, Susumu Inamoto, Seiichiro Kanaya, Yoshiharu Sakai

**Affiliations:** 1 Department of Surgery, Japanese Red Cross Osaka Hospital, Osaka, Osaka, Japan; 2 Department of Gastroenterology and Hepatology, Japanese Red Cross Osaka Hospital, Osaka, Osaka, Japan

**Keywords:** lymphatic fistula, chylous ascites, lymphangiography, indocyanine green, para-aortic lymphadenectomy, lipiodol, high-fat, rectal cancer

## Abstract

**INTRODUCTION:**

Postoperative lymphatic fistula is a relatively rare complication of abdominal and pelvic surgery. Lymphatic fistula is classified based on whether it contains lymphatic ascites with clear lymphatic fluid from the lumbar lymphatic trunks or chylous ascites with milky chyle fluid from the intestinal lymphatic trunk. These two lymphatic trunks eventually converge into the cisterna chyli, which is located at vertebrae T10–L3. In cases of lymphatic fistula following lymphadenectomy in this region, lymphatic leakage from either the intestinal or lumbar lymphatic trunk, or both, should be suspected. Here, we report the successful treatment of a refractory lymphatic fistula after para-aortic lymphadenectomy for rectal cancer by visualizing intestinal lymphatic leakage using enteral high-fat milk and lumbar lymphatic leakage using inguinal intranodal lymphangiography with indocyanine green (ICG).

**CASE PRESENTATION:**

A 57-year-old male developed chylous ascites with elevated triglyceride levels after para-aortic lymphadenectomy. Conservative treatments, including dietary management with fasting, total parenteral nutrition, and administration of octreotide, were ineffective. Although lymphangiography with lipiodol identified lumbar lymphatic leakage, it failed to stop the lymphatic fistula. The intestinal lymphatic leakage site detected by enteral high-fat milk was sutured laparoscopically, and the lumbar lymphatic leakage site was glued with a fibrin sealant patch. However, persistent lymphatic leakage required repeated abdominal paracentesis. Open suturing of the lymphatic leakage site was performed using navigation with ICG and high-fat milk to resolve the lymphatic fistula completely. Lymphatic leakage from the intestinal lymphatic system was detected using enteral high-fat milk and from the lumbar lymphatic trunk using inguinal intranodal lymphangiography with ICG. A total of 5 mL of ICG (1.25 mg/mL) was injected into the inguinal lymph node. ICG leakage was identified at the lumber lymphatic trunk. The leakage site was sutured until the leakage disappeared. Four months after surgery, the ascites disappeared utterly.

**CONCLUSIONS:**

This case demonstrates the efficacy of combining enteral high-fat milk and inguinal intranodal lymphangiography with ICG for accurate detection and differentiation of lymphatic leakage sources. Our dual lymphangiography technique aids in distinguishing leakage from either the intestinal lymphatic or lumbar lymphatic systems, which is critical for the successful treatment of complex lymphatic fistula.

## Abbreviations

ICG
: indocyanine green
IMA
: Inferior mesenteric artery
TPN
: total parenteral nutrition

## INTRODUCTION

Postoperative lymphatic fistula is a relatively rare complication of abdominal and pelvic surgery.^1–3)^ It induces weight gain and abdominal distension due to the intra-abdominal accumulation of ascites. Refractory lymphatic fistula leads to prolonged hospitalization and further debilitation due to electrolyte imbalances and the loss of nutrients and immunoglobulins.^1–2)^

Lymphatic fistula is classified based on whether it contains lymphatic ascites with clear lymphatic fluid or chylous ascites with milky chyle fluid.^1)^ Lymphatic vessels from the lower limbs and pelvic lymphatic system converge into the right and left lumbar lymphatic trunks. Lymphatic fluid from the gastrointestinal organs, which is rich in fat droplets and has a whitish appearance, flows into the intestinal lymphatic trunk. Iatrogenic injury to the lymphatic duct, which includes lymphatic flow from the gastrointestinal organs, induces chylous ascites. These two lymphatic trunks eventually converge into the cisterna chyli, which is located at vertebrae T10–L3.^4,5)^ In cases of lymphatic fistula following lymphadenectomy in this region, lymphatic leakage from either the intestinal or lumbar lymphatic trunks, or both, should be suspected.

We report the successful treatment of a refractory lymphatic fistula after laparoscopic para-aortic lymphadenectomy for rectal cancer by visualizing intestinal lymphatic leakage using enteral high-fat milk and lumbar lymphatic leakage using inguinal intranodal lymphangiography with indocyanine green (ICG).

## CASE PRESENTATION

### Laparoscopic para-aortic lymphadenectomy

**Fig. 1:**
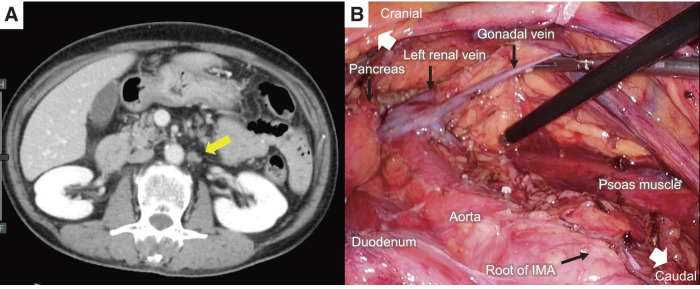
Laparoscopic para-aortic lymphadenectomy for para-aortic lymph node metastasis of rectal cancer. (**A**) Preoperative enhanced computed tomography image showing para-aortic lymph node metastasis (yellow arrow). (**B**) Surgical view after para-aortic lymphadenectomy. IMA, Inferior mesenteric artery

A 57-year-old man was diagnosed with para-aortic lymph node metastasis below the left renal vein 15 months after low anterior resection for rectal cancer (**Fig. 1A**). After 4 months of chemotherapy, a laparoscopic para-aortic lymphadenectomy was performed. The range of dissected para-aortic lymph nodes was between the left renal vein and left common iliac artery craniocaudally, and the aorta and left gonadal vessels transversally (**Fig. 1B**). A vessel-sealing system was used to perform the lymphadenectomy, and the cranial and caudal sides of the lymphatic duct were clipped to prevent lymphatic leakage. Lymph node metastasis was detected histopathologically in one of the harvested lymph nodes. The patient was discharged 5 days after surgery.

### Conservative treatment and lymphangiography using lipiodol

**Fig. 2:**
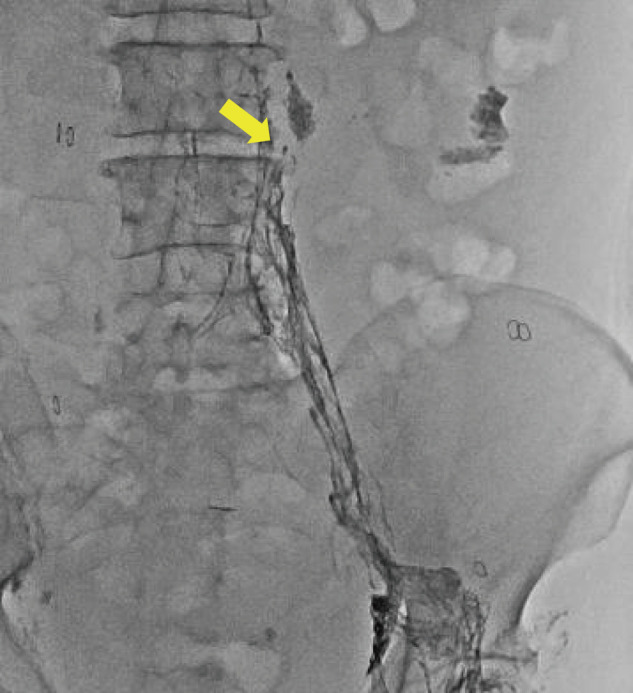
Inguinal intranodal lymphangiography with lipiodol. Lymphatic leakage site from lumbar lymphatic trunk (yellow arrow).

**Fig. 3:**
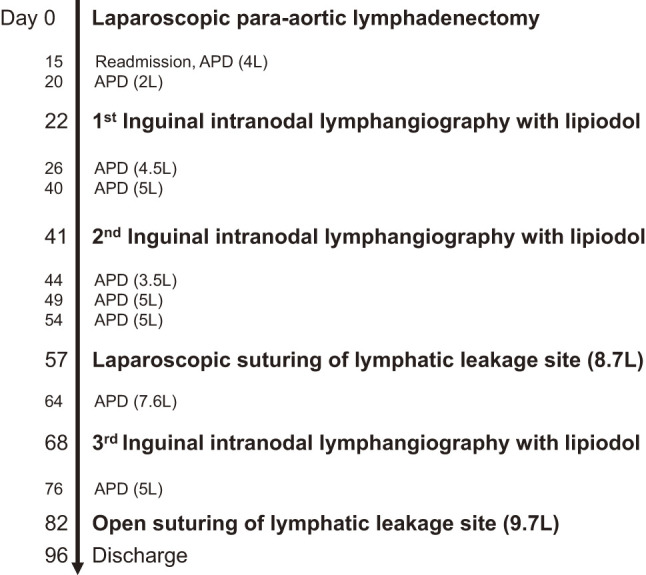
Clinical course and treatment for lymphatic fistula. The amount of drainage ascites is noted in parentheses. APD, Abdominal paracentesis drainage

Two weeks later, the patient was readmitted because of weight gain and abdominal distension. The ascites obtained by abdominal paracentesis had a milky appearance, and analysis revealed a triglyceride concentration of 1155 mg/dL. Conservative treatment for chylous ascites, including dietary management with fasting, total parenteral nutrition (TPN), and the administration of octreotide, albumin, and diuretics, was initiated. However, these treatments did not result in significant improvement. Inguinal intranodal lymphangiography with lipiodol showed leakage on the left side between the third and the fourth lumbar spine (**Fig. 2**). Although we expected the lipiodol to occlude the leakage site, the lymphatic leakage persisted, necessitating repeated abdominal paracentesis for drainage (**Fig. 3**). We suggested two possible causes of this persistent lymphatic leakage: 1) incomplete embolization of the leakage site by lipiodol, and 2) the presence of lymphatic leakage from the intestinal lymphatic system in addition to that from the lumbar lymphatic trunk.

### Laparoscopic suturing of the lymphatic leakage site

**Fig. 4:**
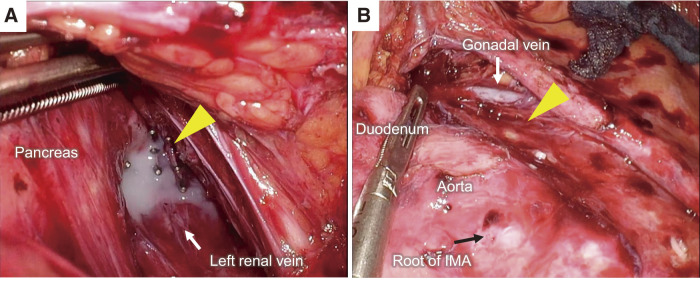
Laparoscopic surgery for refractory lymphatic fistula. (**A**) Chyle lymphatic leakage from the intestinal lymphatic system after enteral high-fat milk (yellow arrowhead). (**B**) Colorless and transparent lymphatic leakage from the lumbar lymphatic trunk (yellow arrowhead). IMA, Inferior mesenteric artery

Laparoscopic suturing of the lymphatic leakage site was performed; 50 mL of high-fat milk was injected into the stomach through a nasogastric tube after induction of anesthesia to detect lymphatic leakage from the intestinal lymphatic system. After the four trocars had been inserted, 8.7 L of milky ascites was drained. Thirty minutes after the injection of high-fat milk, chyle lymphatic leakage was identified behind the pancreas, in front of the left renal vein (**Fig. 4A**). The leakage site was sutured using a nonabsorbable suture, and the leakage disappeared. Colorless, transparent lymphatic leakage from the lumbar lymphatic trunk was also identified on the left side of the aorta above the root of the inferior mesenteric artery (IMA) (**Fig. 4B**). A fibrin sealant patch (TachoSil; Corza Health, San Diego, CA, USA) was glued to this area to prevent iatrogenic sympathetic nerve and aortic injuries. No further lymphatic leakage was observed. Postoperatively, although the weight gain slowed, abdominal paracentesis was still required for drainage to relieve the persistent abdominal distension (**Fig. 3**). Analysis of the ascites revealed a triglyceride level of 45 mg/dL. Inguinal intranodal lymphangiography using lipiodol showed lymphatic leakage at the same location as before surgery. We hypothesized that the lymphatic leakage from the lumbar lymphatic trunk remained unresolved. However, we had to consider the possibility of residual leakage from the intestinal lymphatic system because low triglyceride concentrations might be affected by fasting and TPN.

### Open suturing of the lymphatic leakage site

**Fig. 5:**
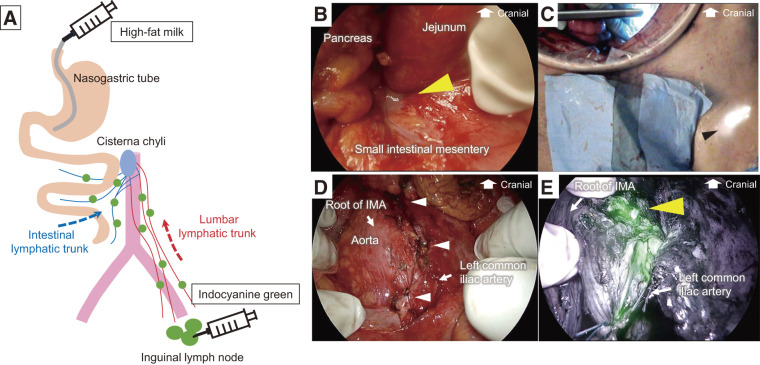
Open surgery for refractory lymphatic fistula. (**A**) Lymphatic leakage from the intestinal lymphatic system was detected using enteral high-fat milk and from the lumbar lymphatic trunk using inguinal intranodal lymphangiography with ICG. (**B**) Chyle lymphatic leakage from the intestinal lymphatic system (yellow arrowhead). **(C)** Left inguinal lymph node after injection of ICG (black arrow). (**D**) Continuous suturing of the para-aortic tissue including the left lumber lymphatic trunk and ligation of the lymphatic duct (white arrowheads). (**E**) Identification of ICG leakage site from the lumbar lymphatic trunk (yellow arrowhead). ICG, Indocyanine green; IMA, Inferior mesenteric artery

Open suturing and ligation of the lymphatic leakage site were performed using navigation with ICG and high-fat milk to completely resolve the lymphatic fistula. Lymphatic leakage from the intestinal lymphatic system was detected using enteral high-fat milk and from the lumbar lymphatic trunk using inguinal intranodal lymphangiography with ICG (**Fig. 5A**).

High-fat milk was injected into the stomach using the same method used for the laparoscopic surgery. Midline laparotomy revealed 9.7 L of milky ascites. Chyle lymphatic leakage had stopped at the laparoscopic suture site. However, chyle lymphatic leakage was still observed behind the pancreas, near the ligament of Treitz, the medial-caudal site of the laparoscopic suture (**Fig. 5B**). After suturing of this site, the chyle lymphatic leakage ceased. The para-aortic tissue, including the left lumber lymphatic trunk, was sutured continuously using a nonabsorbable suture from above the root of the IMA, where the leakage was detected preoperatively on inguinal intranodal lymphangiography using lipiodol, toward the caudal side. Furthermore, the para-aortic lymphatic duct was ligated immediately above and below the left common iliac artery. A total of 5 mL of ICG (1.25 mg/mL) was percutaneously injected into the left inguinal lymph node (**Fig. 5C**). After 80 min, ICG leakage was identified at the suture site using a near-infrared camera (**Fig. 5D** and **5E**). The leakage site was sutured again until the leak had disappeared. Finally, a fibrin sealant patch (TachoSil; Corza Health) was glued to the area. Four months after surgery, the ascites disappeared utterly.

## DISCUSSION

We successfully treated a refractory lymphatic fistula by visualizing intestinal lymphatic leakage using enteral high-fat milk, and lumbar lymphatic leakage using inguinal intranodal lymphangiography and ICG. In lymphatic fistula following para-aortic lymphadenectomy, it is necessary to determine whether the leakage is coming from the intestinal lymphatic system, the lumbar lymphatic trunk, or both. Our dual lymphangiography technique using enteral high-fat milk and ICG was useful for distinguishing between the two anatomically distinct lymphatic drainage systems.

Lymphatic fistula is classified as having lymphatic ascites with clear lymphatic fluid or chylous ascites with milky chyle fluid from the gastrointestinal organ.^1)^ Chylous ascites is diagnosed based on its milky-white appearance and triglyceride concentration exceeding 200 mg/dL.^2,6)^ In this case, the triglyceride concentration was significantly elevated at 1155 mg/dL. Conservative treatment includes 1) diet control (low fat, medium-chain triglyceride diet), fasting, and TPN; and 2) medication (somatostatin analog, diuretics), which is selected as the first-line treatment for chylous ascites.^1,2,6)^ Conservative methods can improve the condition in 66%–77% of patients.^7–10)^ In this patient, although the triglyceride concentration decreased to 279 mg/dL after conservative treatment, the continuous accumulation of chylous ascites could not be prevented.

Lipiodol lymphangiography is usually selected for lymphatic fistula that is refractory to conservative treatment. It is not only a diagnostic tool to identify the lymphatic leakage site, but also a therapeutic tool to induce an inflammatory reaction and occlude the lymphatic duct. The clinical success rate of inguinal intranodal lymphangiography without surgical intervention ranges from 55% to 70%.^11–13)^ However, Alejandre-Lafont et al.^12)^ reported that the success rate decreases when lymphatic drainage exceeds 500 mL/day. Additionally, the lymphatic system from the intestinal organs cannot be visualized through injection into the inguinal lymph nodes. In such cases, mesenteric intranodal lymphangiography is effective in detecting and occluding lymphatic leakage in refractory chylous ascites.^14)^ However, this procedure requires a laparotomy to expose the mesenteric lymph nodes for ultrasound-guided puncture. In our patient, although the lymphatic leakage site was identified through inguinal intranodal lipiodol lymphangiography, the lymphatic fistula could not be controlled because of the high volume of leakage and involvement of lymphatic leakage from the intestinal lymphatic system.

Ligation and suturing of the lymphatic leakage site were performed following the failure of conservative treatment or lymphangiography. Lymphangiography and an enteral high-fat diet are useful for identifying leakage sites. The high-fat diet method, which involves preoperative oral intake or intraoperative enteral administration through a nasogastric tube, is effective in detecting chyle leakage from the intestinal lymphatic system.^15,16)^ Intraoperative lymphangiography with ICG fluorescence has recently been used to detect lymphatic leakage sites. In the current patient, we used the ICG fluorescence method in combination with the enteral high-fat diet method to visualize lymphatic leakage from the lumbar and intestinal lymphatic trunks. However, the optimal injection site, dose, and timing of ICG administration have not been standardized.^17–19)^ ICG was injected into the inguinal lymph nodes in the same way as in inguinal intranodal lipiodol lymphangiography. However, intranodal injection requires skillful technique, and as previously reported, the interdigital or inguinal subcutaneous injections may offer a simpler alternative.^18,20)^ In our case, it took 80 minutes to detect the ICG leakage following ICG injection. Placing the ICG injection site closer to the leakage site, such as the iliac vessel area, may help shorten the detection time.^21)^ In our patient, the ICG injection timing was set after suturing of the lymphatic leakage site, because widespread ICG diffusion from the leakage site may hinder the identification of precise leakage sites and the assessment of complete resolution. When the leakage site has been identified prior to ICG injection, this technique is useful for confirming the disappearance of leakage, a process referred to as the “leak test.”

Initial laparoscopic surgery failed to prevent lymphatic leakage despite the confirmation of its disappearance. A comparison study between laparoscopic and open surgery for lymphatic leakage has not been reported. Minimally invasive procedures have been identified as a risk factor for lymphatic leakage following pancreatic surgery.^22)^ During minimally invasive procedures, CO_2_ insufflation pressure may make lymphatic leakage sites undetectable.^22,23)^ In our case, the residual lymphatic leakage may have been overlooked due to 10 cm H_2_O CO_2_ pressure. The normal in vivo mesenteric lymphatic pressure has not been established. However, the lymph capillary pressure in the leg of healthy volunteers has been reported to be 2.6 ± 2.8 mmHg.^24)^ Considering this finding, reducing the CO_2_ insufflation pressure to approximately 3.5 cm H_2_O may be beneficial. In minimally invasive surgery, after major lymphatic leakage is detected and repaired in our dual lymphangiography, minor lymphatic leakage should be confirmed under low CO_2_ insufflation pressure.

## CONCLUSIONS

We successfully treated refractory lymphatic fistula by examining intestinal lymphatic leakage using enteral high-fat milk, and lumbar lymphatic leakage using inguinal lymphangiography with ICG. This dual lymphangiography technique may be useful for distinguishing between two anatomically distinct lymphatic drainage systems.

## DECLARATIONS

### Funding

No funding was received for this study.

### Authors’ contributions

TO, AN, SY, RK, and SK collected the data and TO wrote the initial draft.

TO designed and edited the manuscript.

RK performed the angiography and AN, SI, SK, and YS advised surgical techniques.

All the authors read and reviewed this paper, and approved the final manuscript.

### Availability of data and materials

Not applicable.

### Ethics approval and consent to participate

The study was approved by the Ethics Committee of the Japanese Red Cross Osaka Hospital (Approval No. 1084), and informed consent was preoperatively obtained from the patient.

### Consent for publication

Informed consent was obtained from the patient for publication of this report.

### Conflict of interest

The authors declare that they have no conflicts of interest.
